# Medical Obituary

**Published:** 1835

**Authors:** 


### XVII.—Obituary.

We copy from the Cherry Valley (New-York) Gazette, of the 24th of March, the following obituary notice. It will be remarked that the deceased was the surgeon who performed an important surgical operation, recorded in the first department of this number.

"Died, on Wednesday, March 18, at his late residence in this town, after a long and distressing illness, which he bore with quiet resignation and unshrinking fortitude, Delos White, M. D. in the 47th year of his age. He was a native of this town. In 1809 he became a graduate of Union College, and immediately after commenced the study of medicine in the city of New York, which he prosecuted with great success. He closed his labors as a student, and began his professional career under the direction and auspices of his father, the late Dr. Joseph White, well known as one of the most eminent medical men in the state. Perhaps no practitioner of the healing art ever possessed more entirely the unwavering confidence of those to whom he was known, (and he was known extensively,) than Dr. Delos White, and certain we are that there are few, if any, who ever better deserved that confidence. He occupied a distinguished station among the first rank of his cotemporaries as a surgical operator, and was for some time a professor of anatomy in the Western Medical College of New York. But independent of the loss of his professional skill and services, in the late bereavement, our society has been deprived of one of its most estimable and useful members—kind and benevolent as a neighbor, and ardent and zealous as a friend, he was loved and is lamented by all. An amiable wife, and a numerous and most interesting family of sons and daughters survive to mourn the irreparable loss of an indulgent and tender parent and affectionate husband.

The funeral of Dr. White on Saturday, was numerously attended. The solemnity and grief which was visible in every countenance, afforded a striking demonstration of the esteem and affection of those who knew how to appreciate his worth and their own loss."

